# Predicting Volume of Distribution in Humans: Performance of In Silico Methods for a Large Set of Structurally Diverse Clinical Compounds[Fn FN5]

**DOI:** 10.1124/dmd.120.000202

**Published:** 2021-02

**Authors:** Neha Murad, Kishore K. Pasikanti, Benjamin D. Madej, Amanda Minnich, Juliet M. McComas, Sabrinia Crouch, Joseph W. Polli, Andrew D. Weber

**Affiliations:** GlaxoSmithKline, Collegeville, Pennsylvania (N.M., K.K.P., J.M.M., S.C., J.W.P., A.D.W.); Lawrence Livermore National Laboratory, Livermore, California (A.M.); Frederick National Laboratory for Cancer Research, Frederick, Maryland (B.D.M.); and Accelerating Therapeutics for Opportunities in Medicine (ATOM) Consortium, San Francisco, California (N.M., K.K.P., B.D.M., A.M., J.M.M., S.C., J.W.P., A.D.W.)

## Abstract

Volume of distribution at steady state (V_D,ss_) is one of the key pharmacokinetic parameters estimated during the drug discovery process. Despite considerable efforts to predict V_D,ss_, accuracy and choice of prediction methods remain a challenge, with evaluations constrained to a small set (<150) of compounds. To address these issues, a series of in silico methods for predicting human V_D,ss_ directly from structure were evaluated using a large set of clinical compounds. Machine learning (ML) models were built to predict V_D,ss_ directly and to predict input parameters required for mechanistic and empirical V_D,ss_ predictions. In addition, log D, fraction unbound in plasma (fup), and blood-to-plasma partition ratio (BPR) were measured on 254 compounds to estimate the impact of measured data on predictive performance of mechanistic models. Furthermore, the impact of novel methodologies such as measuring partition (Kp) in adipocytes and myocytes (*n* = 189) on V_D,ss_ predictions was also investigated. In predicting V_D,ss_ directly from chemical structures, both mechanistic and empirical scaling using a combination of predicted rat and dog V_D,ss_ demonstrated comparable performance (62%–71% within 3-fold). The direct ML model outperformed other in silico methods (75% within 3-fold, *r*^*2*^ = 0.5, AAFE = 2.2) when built from a larger data set. Scaling to human from predicted V_D,ss_ of either rat or dog yielded poor results (<47% within 3-fold). Measured fup and BPR improved performance of mechanistic V_D,ss_ predictions significantly (81% within 3-fold, *r*^*2*^ = 0.6, AAFE = 2.0). Adipocyte intracellular Kp showed good correlation to the V_D,ss_ but was limited in estimating the compounds with low V_D,ss_.

**SIGNIFICANCE STATEMENT:**

This work advances the in silico prediction of V_D,ss_ directly from structure and with the aid of in vitro data. Rigorous and comprehensive evaluation of various methods using a large set of clinical compounds (*n* = 956) is presented. The scale of techniques evaluated is far beyond any previously presented. The novel data set (*n* = 254) generated using a single protocol for each in vitro assay reported in this study could further aid in advancing V_D,ss_ prediction methodologies.

## Graphical abstract

**Figure:**
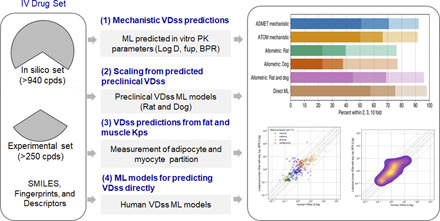

## Introduction

The current drug discovery path is a sequential, time-consuming process with a high attrition rate (Hinkson et al., 2020). Attrition of small-molecule drug candidates due to poor pharmacokinetic (PK) profiles has diminished significantly in recent years (Waring et al., 2015). This advancement can partly be attributed to the unprecedented emphasis on screening compounds based on PK parameters in the drug discovery phase (Ferreira and Andricopulo, 2019). PK is a well recognized and fundamental property that influences drug concentrations at target, which ultimately determines a drug’s efficacy and safety (Ferreira and Andricopulo, 2019). Volume of distribution at steady state (V_D,ss_) is a key PK parameter that describes the relationship between drug concentration measured in plasma or blood to the amount of drug in the body at equilibrium (Smith et al., 2015). Estimation of apparent V_D,ss_ is of utmost importance because it influences *C*_max_ and half-life in plasma and target tissues, which in turn determines dose and dosing regimen in the clinic (del Amo et al., 2013). Toward this end, V_D,ss_ in humans is commonly predicted using preclinical in vivo and in vitro data in conjunction with various allometric scaling methods such as the Oie and Tozer method (Jones et al., 2011). Alternatively, V_D,ss_ can be extrapolated from tissue-to-plasma partition coefficients (Kp) from preclinical species (generally rat) (Nigade et al., 2019). These experiments are resource-intensive and require the synthesis of compounds; these limitations further hinder the ability to predict human V_D,ss_ early in drug discovery or during lead optimization. Thus, considerable effort has been undertaken to develop predictive in silico models to accelerate and reduce the cost of drug discovery processes (Wenzel et al., 2019). As V_D,SS_ is dependent on the tissue partitioning of compounds, numerous studies have focused on developing in silico approaches to predict tissue partitioning based on physicochemical properties such as pKa and log P, plasma protein binding, and blood-to-plasma partition ratio (BPR) (Graham et al., 2012; del Amo et al., 2013). Poulin and Theil were some of the first to propose a mechanistic Kp prediction method (Poulin and Krishnan, 1995; Poulin and Theil, 2002). This method incorporates several important mechanisms, such as albumin binding, neutral lipid, and phospholipid binding. Berezhkovskiy (2004) is another method similar to Poulin and Theil. The Rogers and Rowland method (Rodgers et al., 2005; Rodgers and Rowland, 2006) is by far the most comprehensive Kp prediction method in terms of mechanisms captured. It includes all the mechanisms captured in previous published methods along with the addition of acidic phospholipid and cytosolic ion partitioning. A drawback for the Rogers and Rowland method is that there are two sets of equations based on the dissociation constant or pKa of the compounds, and the cutoff or switch between these equations was set at a pKa of 7. This results in a discontinuous relationship between the dissociation constant and plasma tissue partitioning. Finally, the method is also heavily dependent on accurate pKa predictions. To address these issues, a modified Rodgers and Rowland method was developed (Lukacova et al., 2008) that employs a single continuous combined equation for compounds regardless of pKa. Ion partitioning into acidic or basic intracellular compartments (lysosomes and mitochondria) was described by Trapp et al. (2008) and can be used as an aid to Kp prediction method for compounds for which ion trapping is expected. Key mechanisms that play a crucial role in partitioning itself between plasma and the specific organ tissue implemented by each prediction method is summarized in Table 1.

**TABLE 1:** Comparison of mechanistic tissue partitioning (Kp) prediction methods

Mechanisms	Poulin Homogeneous	Berezhkovskiy	Rogers and Rowland	Lukacova	Trapp Intracellular
Albumin binding	Yes	Yes	Yes	Yes	No
Neutral phospholipid binding	Yes	Yes	Yes	Yes	No
Neutral lipid binding	Yes	Yes	Yes	Yes	No
Acidic phospholipid binding	No	No	Yes	Yes	No
Cytosolic ion partitioning	No	No	Yes	Yes	Yes
Lysosomal ion trapping	No	No	No	No	Yes
Mitochondria ion partitioning	No	No	No	No	Yes
Membrane potential	No	No	No	No	Yes
Intracellular water	Yes	Yes	Yes	Yes	Yes
Extracellular water	Yes	Yes	Yes	Yes	No
Tissue-specific composition	Yes	Yes	Yes	Yes	No

Accurately predicting V_D,ss_ remains a challenge that has not been adequately solved (Smith et al., 2015). Few studies have evaluated the performance of various V_D,ss_ prediction methods; however, these reports were either in preclinical species (Graham et al., 2012) or used a small set (<150) of clinical compounds (Jones et al., 2011; Korzekwa and Nagar, 2017; Chan et al., 2018; Nigade et al., 2019; Mayumi et al., 2020). Recently, Lombardo et al. (2018) published a manually curated data set of V_D,ss_ for 1352 drugs after intravenous dosing, which presented an opportunity to evaluate the predictive performance of various V_D,ss_ methodologies in determining human V_D,ss_. Therefore, we investigated the 1) performance of the most common V_D,ss_ prediction strategies, 2) sensitivity of input parameters that influence V_D,ss_ predictions, 3) impact of experimental data on mechanistic V_D,ss_ predictions, and 4) whether novel methodologies such as using adipocyte and myocyte cell partitioning could improve V_D,ss_ predictions.

## Materials and Methods

### Experimental Approaches

The V_D,ss_ prediction strategies investigated are broadly categorized into two approaches based on the starting data for the analysis, which is either fully in silico (e.g., structural) or in vitro (experimental). Based on the compound availability, an initial in vitro experimental data set of 331 compounds (Lombardo et al., 2018) was identified. Predictive performances were assessed using 956 compounds for the in silico and 254 compounds for the in vitro experimental approaches, respectively.

**Fig. 1.**
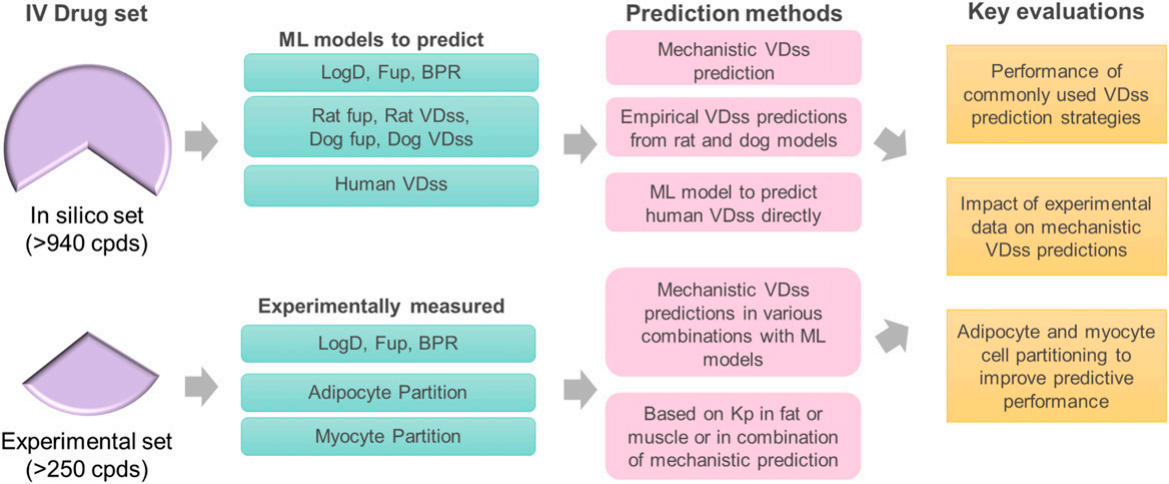
Overview of human V_D,ss_ prediction methods and input parameters (in silico and in vitro data) evaluated in this study.

For the in silico approach, V_D,ss_ was predicted directly from chemical structure [using compound Simplified Molecular Input Line Entry System (SMILES) as input] by using the following four approaches: 1) mechanistic V_D,ss_ prediction using predicted physicochemical properties from commercial software (ADMET Predictor 9.0) or 2) using machine learning (ML) models generated by the Accelerating Therapeutics for Opportunities in Medicine (ATOM) consortium, 3) allometric scaling from predicted V_D,ss_ for preclinical species such as rat and dog ML models, and 4) direct human V_D,ss_ predictions using an ML model built using clinical compounds (see schematic shown in Fig. 1).

In the *Experimental Data* approach, two distinct experimental data sets were generated. The first experimental data set included measurement of physicochemical properties under a single protocol for each in vitro experiment, which included log D, fraction unbound in plasma (fup), and BPR for 331 clinical compounds (Lombardo et al., 2018). The above experimental data were used as input parameters individually or in combination to predict mechanistic V_D,ss_ (Lukacova et al., 2008). In addition, novel experiments were conducted to determine partition of compounds in human adipocytes and myocytes for 200 compounds that were a subset of the 331 compounds selected above. In silico and experimental methodologies are further described in detail below. The percentage of compounds that had accurately predicted V_D,ss_ within 2-, 3-, or 10-fold; *r*^*2*^ (Pearson correlation coefficient); and absolute average fold error (AAFE) were used as key criteria for comparison of predictive performance of each method.

### In Silico Methods

V_D,ss_ of the clinical compounds data set (Lombardo et al., 2018) was subdivided based on whether experimental data were directly measured (331 compounds) or not (970 compounds). Evaluation of in silico methods was performed on both data sets. It is important to note that all the evaluations were performed on a complete hold-out set. For example, when predicting V_D,ss_ for the experimental data set, none of the compounds in the experimental data set were a part of any of the ML model building data sets.

#### ADMET Mechanistic V_D,ss_ Prediction.

ADMET Predictor (version 9.0) was used to predict pKa (S + Acidic_pKa, S + Basic_pKa), fraction unbound in plasma (hum_fup%, converted to fup), BPR, and log P/D (S + log D, S + log P) from chemical structure. These parameters were subsequently used as input parameters to predict mechanistic Kp and human V_D,ss_ predictions (Lukacova et al., 2008). Predicted values of the input parameters were limited to typical assay limits for each of the input parameters (hum_fup%: 0.1%–100%, BPR: 0–200, log P and log D: −3 to 10).

### ATOM Mechanistic, Allometry, and Direct ML Predictions

#### ATOM Mechanistic V_D,ss_ Prediction.

Data sets generated by GlaxoSmithKline (Supplemental Table 1) containing molecular structure information and physicochemical parameters (log D, fup, BPR) were split into train, validation, and test subsets. Model training and evaluation was generally performed as previously described (Minnich et al., 2020). Briefly, a grid search hyperparameter optimization technique was employed to train several machine learning models (neural networks and random forests) with different hyperparameter combinations (learning rate, layer sizes, number of nodes, dropout rates for neural networks and maximum depth, number of trees for random forests), splitting strategies (random and scaffold), and featurization techniques [graph convolution, extended connectivity fingerprint (ECFP), molecular operating environment (MOE) descriptors, and Mordred descriptors]. Additional details related to data sets and model performances are described in Supplemental Table 1. Models with highest validation set *R*^*2*^ (coefficient of determination calculated using sklearn’s *r*^*2*^_score package) regression score function were selected to predict fup, BPR, and log D from chemical structures. These parameters were subsequently used to predict mechanistic Kp and human V_D,ss_ predictions by the Lukacova method (Lukacova et al., 2008) as described in the *ADMET Mechanistic V*_*D,ss*_ *Prediction* section above.

#### Allometric Scaling.

Rat fup, rat V_D,ss_, dog fup, dog V_D,ss_, and human fup values were predicted using ATOM ML models built on GlaxoSmithKline proprietary data sets as described in the *ATOM Mechanistic V*_*D,ss*_ *Prediction* section (Supplemental Table 1). Subsequently, human V_D,ss_ was predicted using the following three methods:

Single-species allometry scaling from rat (Jones et al., 2011)
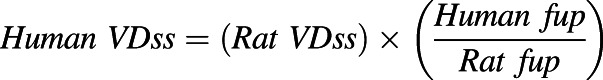
Single-species allometry scaling from dog (Jones et al., 2011)
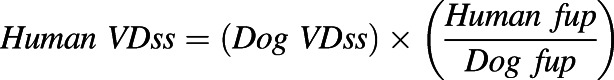
Predicted from rat and dog V_D,ss_ using two species (Wajima et al., 2003)
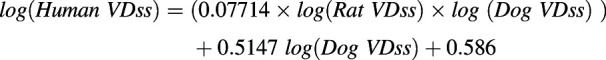

#### Direct ML Models.

An alternative approach to mechanistic prediction of human V_D,ss_ is to build ML models to predict volumes of distribution directly from chemical structures. For this approach, regression models based on molecular structure were fit to directly predict the log base 10 experimental human V_D,ss_ values of clinical compounds (Lombardo et al., 2018). Compounds were clustered by Bemis-Murcko scaffold and subsequently divided into training, validation, and test sets, starting with the largest cluster size to the smallest cluster size. A train/validation/test split of 70%/10%/20% was used to train and evaluate random forest and neural network models as described for the in vitro parameter models (Minnich et al., 2020). Neural network models sampled different combinations of learning rates, layer sizes, and number of nodes. Random forest models sampled different maximum tree depth and number of trees. Several featurization approaches were used including DeepChem’s (https://github.com/deepchem/deepchem) graph convolution model, ECFP, and calculated MOE and Mordred descriptors. Models were selected by picking the model with the maximum validation set *R*^*2*^. Clinical compounds were grouped into two sets. The first set of compounds was the 287 compounds that were selected for experimental measurements (BPR, fup, and log D). The second set of compounds was the 970 additional compounds described in Lombardo et al. (2018) without further experimental measurements. These sets were used in two ways for fitting and prediction. 1) To compare predictive performance of the direct ML models against the other in vitro approaches, models were trained using the 970 human V_D,ss_ of compounds without further experimental measurements. The V_D,ss_ ML model was then used to predict V_D,ss_ for the 287 compounds with new experimental measurements for comparison with in vitro methods. 2) A very challenging (due to the small size of the training set) external test set was used by inverting the previous approach. Models were developed using 287 compounds with new experimental measurements. Then, the fit model was used to predict V_D,ss_ for the 970 compounds without further experimental measurements. In both approaches, the set of compounds used for model development was further split into training, validation, and internal test sets as previously described.

### Experimental Data

#### Log D.

The chromatographic hydrophobicity index (CHI) (Valkó et al., 1997) values were measured using a reversed phase high-performance liquid chromatography (HPLC) column (50 × 2 mm 3 µM Gemini NX C18; Phenomenex, UK) with fast acetonitrile gradient at starting mobile phase of pH 2, 7.4, and 10.5. CHI values are derived directly from the gradient retention times using calibration parameters for standard compounds. The CHI value approximates to the volume percent organic concentration when the compound elutes. CHI is linearly transformed into ChromlogD (Young et al., 2011) by least-squares fitting of experimental CHI values to calculated ClogP values for over 20,000 research compounds using the following formula: ChromlogD_pH=7.4_ = 0.0857CHI-2.00.

#### Blood-to-Plasma Partition Ratio.

In vitro measurement of blood-to-plasma partition was conducted in human blood (K_2_EDTA as anticoagulant) obtained from a commercial source (BioReclamation IVT, Liverpool, NY). Hematocrit (the ratio of volume of red blood cells to total blood) was measured by centrifugation of the whole blood at 3000 rpm for 10 minutes using microhematocrit capillary tubes. Control plasma was prepared from a portion of the whole blood by centrifugation at 3000*g* for 10 minutes. Both whole blood and control plasma samples were warmed at 37°C in a water bath for 30 minutes. Subsequently, the test compounds (1 µM in the final concentration) and controls [methazolamide (BPR ∼1) and metoprolol (BPR ∼40)] were spiked into blood and incubated at 37°C (5% CO_2_) with shaking at 200 rpm for 60 minutes along with control samples. After incubation for 60 minutes, the incubated whole blood was removed from the water bath, and the plasma was separated by centrifugation at 1000*g* for 10 minutes. Aliquots of the control plasma were also removed. All plasma samples (50 µl) were treated with 400 µl of ice-cold acetonitrile containing an internal standard (100 ng/ml tolbutamide in acetonitrile). After the removal of protein by centrifugation at 1640*g* (3000 rpm) for 10 minutes at 4°C, the supernatants were transferred to HPLC autosampler plate. Test compounds and internal standard response (or peak area) ratio in whole blood and its resulting plasma were measured using liquid chromatography with tandem mass spectrometry (LC/MS/MS). Blood-to-plasma partition was calculated by ratio of mass spectrometric response of compounds in blood samples after 60 minutes of incubation to mass spectrometric response in plasma samples.

#### Fraction Unbound in Plasma.

In vitro measurement of fup was conducted using a rapid equilibrium dialysis (RED) device. The fup values of test compounds and a positive control (warfarin) were determined at a single time point of 4 hours postincubation. Considering high surface-to-volume ratio of the membrane compartment in a RED device, equilibrium is expected to be achieved within 4 hours of incubation (Waters et al., 2008). Stock solutions of test compounds and warfarin were prepared in DMSO at concentrations of 5 mM and subsequently diluted to a final concentration of 0.5 mM in DMSO:water (1:1, v/v). Incubation mixtures were prepared by diluting the stock solution into human plasma obtained from a commercial source (BioReclamation IVT). Final concentrations of compounds in incubation mixture were 5 µM. Human plasma was prewarmed in a water bath at 37°C prior to the experiment. In total, 400 µl of the stopping solution (100 ng/ml tolbutamide in acetonitrile) was added to a 96-well deep well sample collection plate on ice. In a RED device, 500 µl of PBS was added to the white chambers (receiver side), and aliquots (300 µl) of each incubation mixture were spiked into the red wells (donor side). A sample (40 µl) of the incubation mixture was transferred into the 0-minute wells on the sample collection plate. The device and remaining spiked plasma samples were incubated at 37°C for 4 hours with shaking at 150 rpm. After the incubation period, 40 µl of the remaining spiked plasma was transferred to the sample collection plate. All samples in the RED device were mixed by pipetting prior to aliquoting (40 µl) from each donor well into a well containing 160 µl of PBS buffer. A sample (160 µl) of each receiver well was aliquoted into a tube containing 40 µl of blank plasma. PBS (160 µl) was added to the 0-minute and 240-minute stability wells. Analysis of samples was performed using LC/MS/MS. For all samples, peak area ratios were used to determine percent unbound. Plasma proteins were precipitated with 400 μl of acetonitrile containing 100 ng/ml tolbutamide as a mass spectral internal standard. The resulting mixtures were vortex-mixed, followed by centrifugation for 15 minutes at >3500 rpm/min. A sample (100 µl) of the supernatant/well was transferred to a clean 96-well plate containing 100 µl of ultrapure water/well. The plate was vortexed for 1 minute at >1700 rpm/min. Aliquots (4 µl) of the resulting supernatant were injected onto the LC/MS/MS system to obtain peak area ratios for each compound to determine fraction unbound in plasma. Equilibrium dialysis method for measuring fup is amenable to automation and is generally accepted as the gold standard (Trainor, 2007).

#### Adipocyte and Myocyte Partition.

Intracellular partition of compounds in adipocytes and myocytes was determined using a protocol described previously (Treyer et al., 2018). Primary human adipocytes and myocytes were obtained from commercial sources (Lonza, MD). The test compounds and controls at a final concentration of 0.5 μM were incubated with fully differentiated myocytes and adipocytes plated in culture in triplicate at 37°C (5% CO_2_) with shaking at 100 rpm for 45 minutes. After the end of the incubation, the medium was transferred to a stop solution containing acetonitrile and internal standard (100 ng/ml tolbutamide in acetonitrile). The cell layer was washed with 200 µl of cold Hanks’ buffered salt solution and extracted with stop solution (100 ng/ml tolbutamide in acetonitrile). Both the intracellular and extracellular compound concentrations were analyzed using LC/MS/MS. The cell protein concentration was determined by the bicinchoninic acid assay. Intracellular drug accumulation (Kp) was calculated from the peak area ratios of the analyte to internal standard in the medium, cells, and protein concentration from the following 
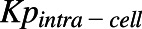
 equation. Protein content was quantified using the bicinchoninic acid assay in representative wells to calculate the cellular volume (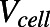), assuming 6.5 μl/mg protein (Treyer et al., 2018). Amount of drug in the cells 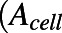) was estimated using peak area ratio and volume of cell lysate (area ratio × volume of cell lysate). 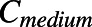 refers to corrected medium concentration. Intracellular accumulation was determined using cell lysate concentration × volume of cell lysate (150 µl). Subsequently, the 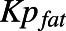 or 
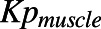
 is calculated accounting from protein binding in plasma.
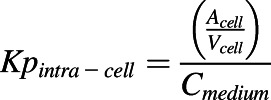

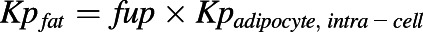

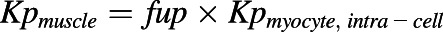


### Predictions Based on Experimental Data

#### Mechanistic Models for Kp Prediction.

Experimental data (log D, fup, BPR) were used as input parameters individually or in combination to predict Kp (Lukacova et al., 2008) and subsequently were used to calculate V_D,ss_ using the following relationship:
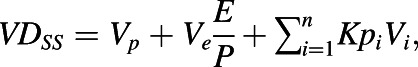
where 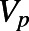 is the volume of plasma; 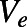 is the volume of erythrocytes (
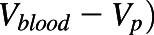
; *E*/*P* is the erythrocyte-to-plasma ratio, which is derived by the equation BPR + hematocrit − 1)/hematocrit; and 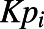 and 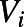 are the plasma tissue partition ratio and volume, respectively, for the 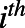 tissue (Nigade et al., 2019).

#### Tissue-Level Kp Prediction.

We used five strategies for predicting V_D,ss_ using adipocytes and myocyte Kp values:

1. Adipocyte-only method: Adipocyte Kp values were used to calculate partitioning into fat (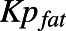). Kp for other organs was assumed to be 1 to predict V_D,ss_ using the following equation:
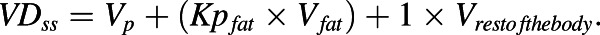
2. Myocyte-only method: Myocyte Kp values were used to calculate partitioning into muscle tissue (
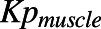
), and Kp for other organs was assumed to be 1 to predict V_D,ss_ using the following equation:
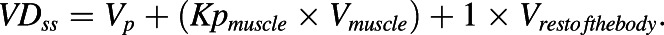
3. Combined method: Both adipocyte and myocyte Kp values were used to calculate fat and muscle volumes, respectively. Kp for all nonfat and muscles organs was assumed to be 1 to predict V_D,ss_.
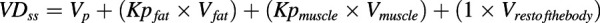
4. Average method: Average of adipocyte and myocyte Kp values were used as Kp for all nonfat and muscle tissues. Both adipocyte and myocyte Kp values were used to calculate fat and muscle volumes, respectively, to predict V_D,ss_.
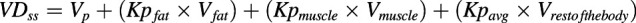
5. Separate method: Mechanistic Kp (Lukacova et al., 2008) calculations were used for nonfat or nonmuscle organs. Both adipocyte and myocyte Kp values were used to calculate fat and muscle volumes, respectively. Both of the volumes were subsequently added to predict V_D,ss_ as follows:
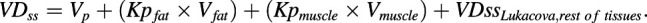

## Results

As summarized in Fig. 1, we investigated the performance of the most common V_D,ss_ prediction strategies, sensitivity of input parameters that influence V_D,ss_ predictions, impact of experimental data on mechanistic V_D,ss_ predictions, and whether adipocyte and myocyte cell partitioning could improve predictive performance by using a large compound data set. An in silico–only approach was applied using a set of 956 compounds (the ATOM in silico set) related to the Lombardo intravenous dosing drug set (*n* = 1352 drugs) in which V_D,ss_ values were reported (Lombardo et al., 2018). A separate set of compounds, the ATOM experimental set (*n* = 254 compounds), had additional in vitro data collected under uniform experimental conditions (see *Materials and Methods*; Supplemental Table 2) and was used as a comparator against the purely in silico methods. Although the ATOM experimental data set was selected based on the compound availability from an initial set of 331 drugs, it represented chemical diversity of the clinical data set (Supplemental Fig. 1).

**Fig. 2.**
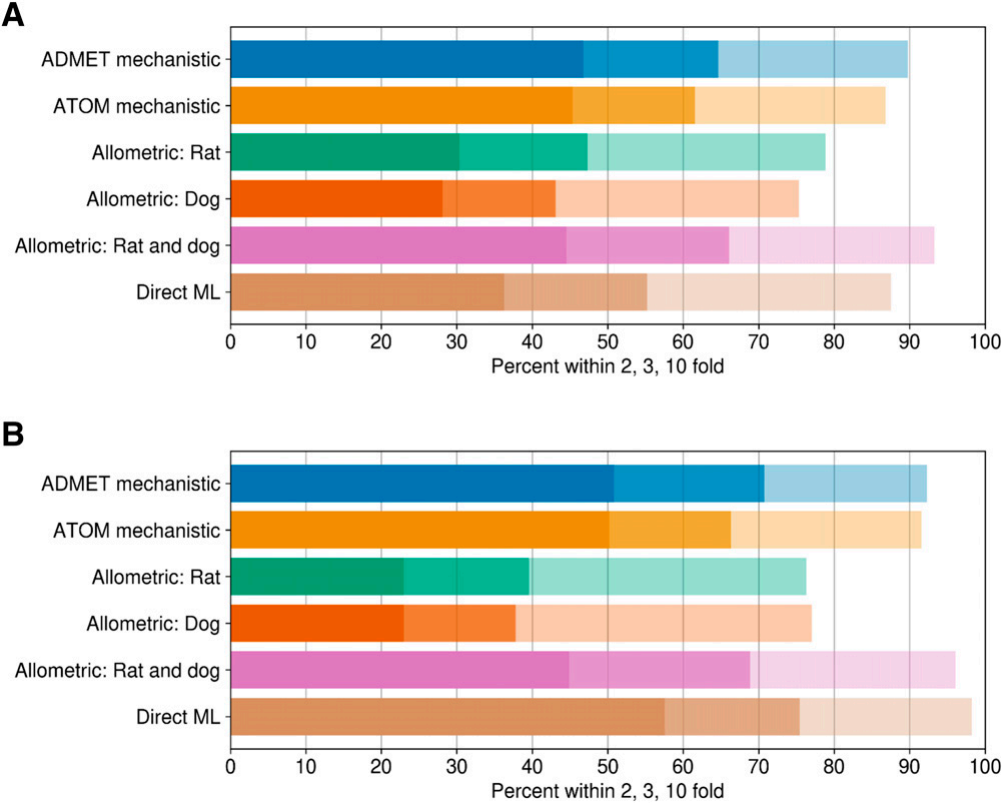
Summary of model performance of in silico V_D,ss_ prediction methodologies: (A) ATOM in silico set (*n* = 956 compounds) and (B) ATOM experimental set (*n* = 254 compounds).

**Fig. 3.**
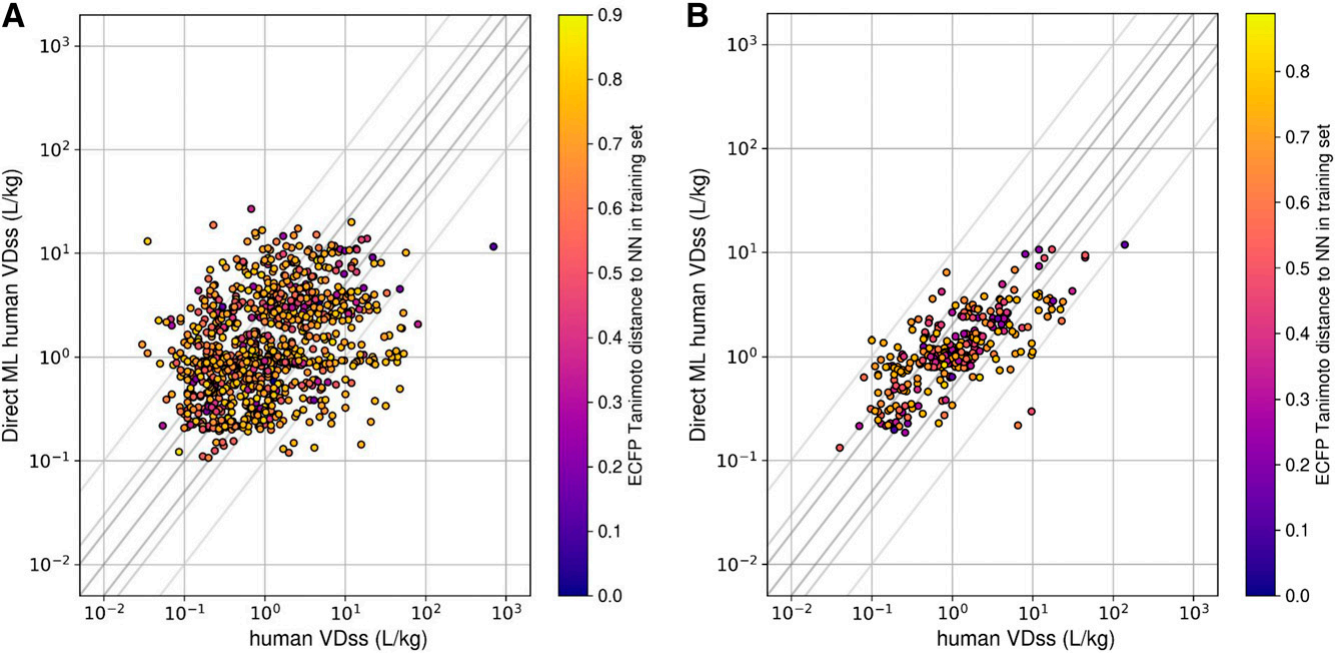
Predicted vs. observed V_D,ss_ using direct ML models: (A) the ML model built was using a smaller data set (287 compounds), and predictions were tested on a large in silico set (956 compounds) and (B) vice versa. Crosslines indicate 2-, 3-, and 10-fold limits.

The comparative assessments of various in silico approaches evaluated to predict human V_D,ss_ for two discrete sets of compounds are summarized in Fig. 2 and Table 2. Details of ATOM ML models used to predict input parameters for mechanistic V_D,ss_ predictions are shown in (Supplemental Table 1). Model/featurization combination that resulted in the best models varied by data sets. MOE or graph convolution featurization with random forest or neural network models most frequently outperformed other featurization and models investigated in this study. Relative to other in silico methods, mechanistic V_D,ss_ predictions (both by ATOM and ADMET ML models) and two-species allometry demonstrated superior predictive performance, with 62%–71% of compounds within 3-fold of observed V_D,ss_ for both data sets (Table 2). In contrast, scaling from single species using allometric methods performed poorly, with only 38%–47% of compounds within 3-fold (Table 2). Trends in predictive performance (such as percentage within 2-, 3-, and 10-fold; AAFE; and Pearson’s *r*^*2*^) across various in silico models were comparable using either the smaller or larger data sets (Table 2, 283 and 956 compounds), with an exception for direct ML model. Predictive performance of the direct ML model to predict V_D,ss_ increased significantly when the ML model was built using a larger data set (Fig. 3; Table 2). The percentage of compounds within 2-, 3-, and 10-fold increased to 58%, 75%, and 98% from 36%, 55%, and 88%, respectively (Fig. 2; Table 2). Similarly, there was significant improvement in *r*^*2*^ values (from 0.14 to 0.52) and AAFE (decreased from 3.3 to 2.2). The scatter plots of direct ML model predictions are shown in Fig. 3. Additional scatter plots of predicted V_D,ss_ compared with reported (Lombardo et al., 2018) values across both data sets and various in silico methods are presented in Supplemental Fig. 2.

**TABLE 2:** Summary of model performance of in silico V_D,ss_ prediction methodologies for Lombardo intravenous dosing drug set (*n* = 1352 drugs) divided into two subsets: 1) ATOM in silico set (>940 compounds) and 2) ATOM experimental set (*n* > 280 compounds)

Method Description	Input Parameters	*n*	Within 2-Fold	Within 3-Fold	Within 10-Fold	*r*^*2*^	AAFE
			*%*				
ADMET mechanistic	Log D, fup, BPR (predicted using ADMET Predictor models)	956	47	65	90	0.25	2.8
		287	51	71	92	0.35	2.4
ATOM mechanistic	Log D, fup, BPR (predicted using ATOM ML models)	936	45	62	87	0.23	3.1
		285	50	66	92	0.38	2.7
Allometry (rat and dog)	Rat V_D,ss_, dog V_D,ss_ (predicted using ATOM ML models)	956	45	66	93	0.28	2.7
		283	45	69	96	0.37	2.5
Allometry (rat)	Rat V_D,ss_, rat fup, fup (human) (predicted using ATOM ML models)	956	30	47	79	0.02	4.4
		283	23	40	76	0.0	4.9
Allometry (dog)	Dog V_D,ss_, dog fup, fup (human) (predicted using ATOM ML models)	956	28	43	75	0.05	4.9
		283	23	38	77	0.12	4.7
Direct ML model for human V_D,ss_	SMILES/MOE descriptors	956	36	55	88	0.14	3.3
		285	58	75	98	0.52	2.2

**Fig. 4.**
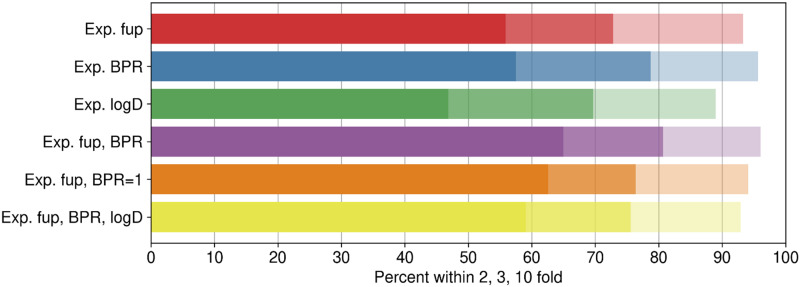
Predictive performance of mechanistic Kp prediction methods using various combinations of experimental (Exp.) data.

Experimentally measured log D, fup, and BPR in vitro assays for 254 compounds are summarized in Supplemental Table 2. Although 331 compounds were originally included, some of the compounds showed analytical or recovery issues in different assays and were removed from the data sets. Figure 4 and Table 3 summarize predictive performance of various combinations of experimental data (Supplemental Table 2) as input parameters. Scatter/kernel density estimation plots of mechanistic V_D,ss_ predictions using various combinations of experimental data (fup, BPR, and log D) as input parameters are shown in Supplemental Fig. 4. The highest percentage of compounds within 3-fold of prediction error was observed when experimentally determined fup and BPR were used as input parameters, with 81% of the compounds within 3-fold of Lombardo reference values; a good correlation between predicted and observed values (*r*^*2*^ = 0.58) was seen.

**TABLE 3:** Summary of mechanistic V_D,ss_ predictive performance using experimental data (fup, BPR, and log D) as input parameters

Method Description	Input Parameters	*n*	Within 2-Fold	Within 3-Fold	Within 10-Fold	*r*^*2*^	AAFE
			*%*				
Experimental fup	Experimental fup. Other input parameters were predicted by ATOM ML models	254	56	73	93	0.42	2.3
Experimental BPR	Experimental BPR. Other input parameters were predicted by ATOM ML models	254	57	79	96	0.51	2.1
Experimental log D	Experimental log D. Other input parameters were predicted by ATOM ML models	254	47	70	89	0.29	2.7
Experimental fup, BPR	Experimental fup and BPR. Other input parameters were predicted by ATOM ML models	254	65	81	96	0.58	2.0
Experimental fup, BPR, log D	Experimental fup, BPR, and log D	254	59	76	93	0.46	2.2
Experimental fup (BPR = 1)	Experimental fup, and BPR is assumed equal to 1. Other input parameters were predicted by ATOM ML models	254	63	76	94	0.48	2.1

**Fig. 5.**
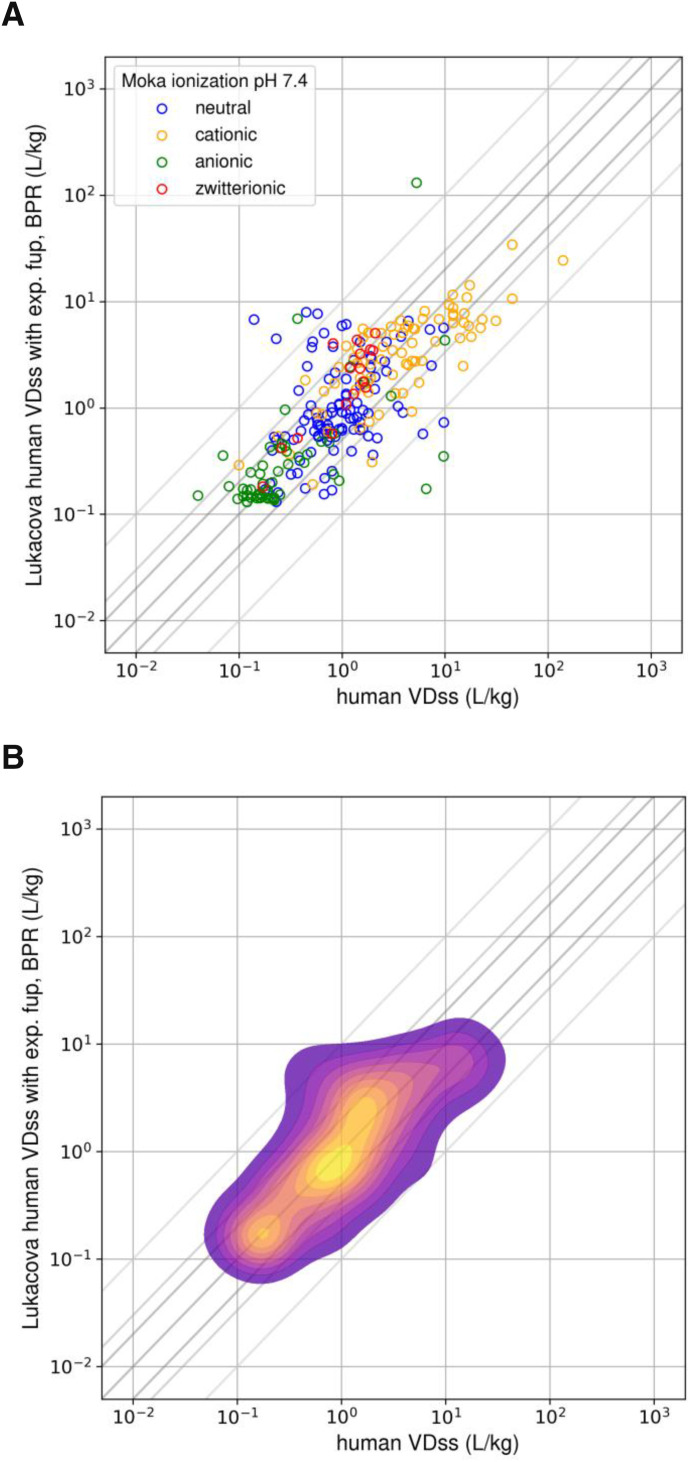
(A) Scatter plot [colored by ionic state reported in Lombardo et al. (2018)]. (B) Kernel density plot showing correlation between observed and predicted V_D,ss_ for 254 compounds using experimental (exp) fup and BPR data as input parameters. Crosslines indicate 2-, 3-, and 10-fold limits.

Correlation between observed and predicted V_D,ss_ for 254 compounds using experimental fup and BPR data as input parameters is shown in Fig. 5. Among the experimental parameters investigated, V_D,ss_ predictions were sensitive to BPR. V_D,ss_ predictions within 3-fold dropped to 73% from 81%, and *r*^*2*^ reduced from 0.58 to 0.42 when only fup was used instead of fup and BPR. In absence of experimental data, assuming BPR as 1 could be recommended, as better performance was observed when the BPR value was assumed to be 1 instead of inputting ML-predicted values (Table 3); 63% of the compounds were predicted within 2-fold when BPR was assumed to be 1, compared with 56% when BPR was predicted from ML models in combination with measured fup. This highlights that V_D,ss_ predictions are sensitive to errors in BPR predictions from ML models and that the best performance across all the methods is with measured fup and BPR values. In contrast, complementing measured log D to mechanistic predictions with fup and BPR measured data did not improve predictive performance any further (Table 3). Since predicted values from log D ML models (both ADMET and ATOM) were in close agreement with measured values (Supplemental Fig. 3), it is not surprising to see that measurement of log D values did not improve V_D,ss_ predictions. Figure 5A displays the correlation of predicted-to-observed V_D,ss_ classified by ionization class (Lombardo et al., 2018). Anionic and zwitterionic compounds are the best-predicted classes compared with neutral compounds. The kernel density estimation (Seaborn Python library: https://seaborn.pydata.org/tutorial/distributions.html) plot in Fig. 5B demonstrates underlying distribution of the points in the Fig. 5A scatter plot. Figure 5B suggests that overall predictions using mechanistic predictions using measured fup and BPR are directly correlated, and a majority of the predictions are on the unity line, highlighting that there is no overall trend of overpredicting or underpredicting V_D,ss_.

**Fig. 6.**
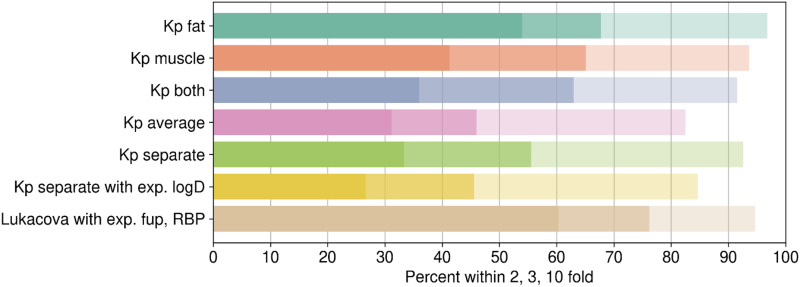
Predictive performance using adipocyte (Kp fat) and myocyte (Kp muscle) partitioning experimental (exp) data.

As fat and muscle contribute to 60% of body volume, the impact of experimental adipocyte and myocyte cell partition in improving V_D,ss_ prediction was investigated. Measured intracellular partitioning of 189 compounds in adipocytes and myocytes is presented in Supplemental Table 3. The impact of adipocyte and myocyte cell partition on predictive performance for the same set of compounds was compared with that from the best predictive model (fup and BPR experimental data as input parameters; Fig. 6; Table 4). Good correlation between observed versus predicted V_D,ss_ was noted when either adipocyte or myocyte or both Kp values were used (*r*^*2*^ of 0.41–0.48, Table 4). Although the percentage of compounds within 3-fold, *r*^*2*^, and AAFE were not significantly different using either adipocyte or myocyte partitioning, percentage of compounds within 2-fold was significantly higher when V_D,ss_ was predicted using adipocyte Kp values (54% vs. 41%, Table 4). The combination of both adipocyte and myocyte partitioning with different strategies did not improve predictive performance any further (Table 4). For the same set of compounds, V_D,ss_ predicted using only fup and BPR experimental data demonstrated higher percentage of compounds with 2- and 3-fold compared with predictions based on adipocyte or myocyte data (Fig. 6; Table 4).

**TABLE 4:** Performance of V_D,ss_ prediction methods utilizing adipocyte and myocyte Kp experimental data

Method Description	Input Parameters	*n*	Within 2-Fold	Within 3-Fold	Within 10-Fold	*r*^*2*^	AAFE
			*%*				
Kp fat only	Kp adipocyte, fup and BPR	189	54	68	97	0.42	2.4
Kp muscle only	Kp myocyte, fup and BPR	189	41	65	94	0.43	2.6
Kp fat and muscle	Kp adipocyte, Kp myocyte, fup and BPR	189	36	63	92	0.46	2.9
Kp average of fat and muscle	Kp adipocyte, Kp myocyte, fup and BPR	189	31	46	83	0.46	3.9
Kp fat and Kp muscle with mechanistic predicted Kp values for other tissues	Kp adipocyte, Kp myocyte, fup and BPR, predicted Kp values using ATOM mechanistic models for tissues other than fat and muscle	189	33	56	93	0.48	3.1
Kp fat and Kp muscle with mechanistic predicted Kp values for other tissues using experimental log D	Kp adipocyte, Kp myocyte, fup and BPR, predicted Kp values using ATOM mechanistic models for tissues other than fat and muscle	169	27	46	85	0.41	3.9
Lukacova with experimental fup, BPR	Experimental fup and BPR. Other input parameters were predicted by ATOM ML models	189	60	76	95	0.50	2.2

Across all the prediction methods evaluated using different data sets, there was a good correlation between AAFE and percentage of compounds within 2- or 3-fold of observed. As anticipated, prediction methods in which lower AAFEs were observed demonstrated the highest percentage of compounds within 3-fold. Among all the methods investigated, mechanistic V_D,ss_ predictions utilizing measured fup and BPR as input parameters demonstrated superior performance, with lowest AAFE, highest *r*^*2*^, and percentage of compounds within 3-fold.

## Discussion

### 

#### Mechanistic V_D,ss_ Predictions.

Kp calculations use physiologic parameters of the tissue and physicochemical properties of the drug to ascertain how compounds partition themselves between plasma and tissue. Based on preliminary evaluations and other reports in the literature (Graham et al., 2012), the Lukacova method (Lukacova et al., 2008) was used as a method of choice for mechanistic V_D,ss_ predictions. Key prerequisite input parameters to predict mechanistic V_D,ss_ are pKa, log D, log P, fup, and BPR. Therefore, estimating these input parameters either by in silico methods or by experimental measurements, and impact of measured parameters on mechanistic V_D,ss_ predictions have been explored.

Mechanistic V_D,ss_ predictions using input parameters predicted by either ATOM ML models or ADMET Predictor demonstrated similar performance across data sets (Table 2). Therefore, either of the two ML models set (ATOM or ADMET Predictor) can be used to predict mechanistic V_D,ss_ in silico. It is important to note that ML models for BPR [ATOM ML or ADMET Predictor (from user manual)] were built using very small data sets (Supplemental Table 1), and predictive performances of ML models to predict BPR are questionable. When predicted BPR values were replaced with experimental data, significant improvement in mechanistic V_D,ss_ predictive performance was observed; *r*^*2*^ increased from 0.38 to 0.51 and percentage within 3-fold increased from 66% to 79%, highlighting the sensitivity of V_D,ss_ predictions to BPR values (Fig. 4; Table 3). As BPR is a key parameter, particularly for calculation of intracellular acidic phospholipid binding of strongly basic drugs, it could be anticipated to improve the predictions. However, impact of BPR measurement was not definitely demonstrated in literature until recently (Yau et al., 2020). The current evaluations (Table 3) clearly demonstrate the importance of measuring BPR in predicting V_D,ss_ and the need to fill the existing gaps in BPR data sets used to build predictive ML models. It is noteworthy that with only two in vitro measurements (fup and BPR), 81% of compounds are within 3-fold of observed V_D,ss_ (Table 3), with AAFE of 2.0.

Because it can impact both the pharmacokinetics and pharmacodynamics of a drug, fup is measured routinely in drug discovery (Smith et al., 2010). On the other hand, BPR of compounds in the early discovery phase is relatively less routinely measured and might lead to missed opportunities not only in predicting V_D,ss_ (as observed in this study) but also in predicting the impact on overall pharmacokinetics of a compound (Kalamaridis and DiLoreto, 2014). Comparable predictive performance was noted by Chan et al. (2018) using a smaller data set of 152 clinical compounds. They demonstrated that mechanistic V_D,ss_ predictions were accurate or superior to empirical approaches based on the extrapolation of V_D,ss_ from preclinical species (Chan et al., 2018). In addition to superior performance of mechanistic V_D,ss_ prediction methods (using either ML-predicted or experimental input parameters), a mechanistic approach uniquely offers the ability to calculate partitioning (Kp) of compounds into various tissues.

#### Allometric Scaling.

Traditionally, prediction of human V_D,ss_ has relied on scaling of V_D,ss_ obtained from preclinical species using allometric equations (Jones et al., 2011). Although allometry has some limitations in predicting distribution of highly protein-bound drugs, it has been a valuable technique to predict human PK parameters to determine first-time-in-human dose (Choi et al., 2019). To leverage existing data from animal studies during early drug discovery, use of ML-predicted V_D,ss_ employing allometric scaling from preclinical species was explored. Although there continue to be translational questions about interspecies scaling, it was hypothesized that deployment of this technique could allow for much wider chemical space coverage relative to human V_D,ss_ trained models, as well as to provide insight into mechanisms not captured by mechanistic models such as transporter-driven tissue uptake. Although ML models to predict V_D,ss_ and fup values in preclinical species have demonstrated good performance (Supplemental Table 1), single-species scaling performed poorly in predicting human V_D,ss_ (Table 2, <50% were within 3-fold). This poor performance could be due to magnification of errors in predictions of V_D,ss_ and/or fup values in addition to limitations of single-species scaling. Several studies have shown that plasma protein binding corrections significantly enhanced predictive performance of allometric scaling from preclinical V_D,ss_ (Zou et al., 2012). As the V_D,ss_ predictions are inversely proportional to fup in preclinical species (see *Materials and Methods* for equations), errors in the predictions of fup values will have a significant impact on V_D,ss_ predictions. Therefore, we investigated V_D,ss_ comparisons without fup corrections. Direct correlation of predicted dog V_D,ss_ (without fup corrections) with human V_D,ss_ demonstrated improved performance, with 48%, 65%, and 97% of compounds within 2-, 3-, and 10-fold of observed human V_D,ss_, respectively, when compared with fup accounting for the difference between dog and human (23%, 37%, and 75%, Table 2). This supports that the poor predictive accuracy of the dog fup model magnified the prediction errors. However, similar improved performance or correlations were not observed in the case extrapolating from rat V_D,ss_ predictions. In contrast, human V_D,ss_ scaled using both rat and dog by the Wajima method demonstrated predictive performance similar to mechanistic models (Table 2). Although overall predictive performance is not significantly different between the two methods, it is noteworthy that mechanistic models were relatively better at predicting anionic compounds within 2-fold compared with the Wajima method (Supplemental Fig. 7). V_D,ss_ predictions classified by ionization class across various methods can be found in Supplemental Fig. 6.

#### Direct ML Models.

Previously, we observed that the data set size has a direct impact on model predictivity for several pharmacokinetic related data sets (Minnich et al., 2020). As anticipated, ML models built using smaller data sets, such as that for BPR, showed lower model performance statistics compared with models built using a larger data set (Supplemental Table 1). Furthermore, the direct ML model built on a larger data set (using 970 clinical compounds) outperformed other in silico methods, including the mechanistic V_D,ss_ method (Table 2). When utilizing direct ML models built on a larger data set, 75% of compounds (Table 2) were predicted within 3-fold of observed V_D,ss_, with excellent correlation (Fig. 3B). It is important to highlight that the clinical data set is highly diverse across physicochemical, in vitro ADME, and in vivo PK properties (Lombardo et al., 2018). Models built on diverse data sets of chemical space have a greater applicability domain and generalizability (Simeon et al., 2019). Therefore, direct ML predictions of V_D,ss_ might be the most computationally efficient and predictive way to process in silico predictions of V_D,ss_ for de novo compounds. One limitation of the current model is the relatively small training set, possibly restricting the application of the model to certain chemotypes. In such cases, models that are limited to structurally related analogs may prove more predictive than global models built on a diverse set of compounds (Simeon et al., 2019). Despite some differences in hyperparameters and data set splits used relative to our study, Simeon et al. (2019) demonstrated similar predictive performance for a direct ML model built using a data set of 941 compounds. These independent studies provide promising evidence of improved performances of direct ML models with enhanced data sets of clinical compounds.

#### Predictions Using Adipocyte and Myocyte Cell Partitioning.

Muscle and fat are tissues with larger physiologic volumes (60% of tissue volume), and distribution of compounds to these tissues have a major impact on the V_D,ss_ of compounds in human (Davies and Morris, 1993). Björkman (2002) evaluated relative contributions of various tissue partition coefficients (Kp, tissues) in predicting V_D,ss_ in rat and observed an excellent linear correlation (>0.99) between V_D,ss_ when calculated using only Kp values from muscle and fat. In this study, we hypothesized that intracellular partitioning of compounds into human adipocytes and myocytes in vitro could be used as a surrogate to determine fat and muscle Kp values and subsequently be used to estimate human V_D,ss_. In addition, measuring Kp values directly in human cells could improve translation to human tissues. Higher predictive performance was observed, but only when one of the adipocyte partition or myocyte partition values was included to predict V_D,ss_ (Table 4). Adipocyte and myocyte partition values and predicted V_D,ss_ were highly correlated (*r*^*2*^ > 0.7), suggesting that measurement of partition in only one cell type is adequate. Between the two measurements, adipocyte partition (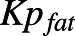
only) showed better performance, particularly with respect to the percentage of compounds within 2-fold compared with myocyte partition (Kp muscle only). Combination of both adipocyte and myocyte partition in various combinations did not provide significant improvement in V_D,ss_ predictions (Table 4). Although, 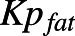 showed good correlation to human V_D,ss_, it failed to predict compounds with low V_D,ss_ (<1 l/kg) because of volume contributions from other tissues (assumption of Kp = 1) (Supplemental Fig. 5a). Surprisingly, predictive performance was lower when fat and muscle volumes were predicted using both adipocyte and myocyte measured data, and the volume of the remaining tissues was predicted using mechanistic Kp prediction method. Only 56% of the compounds were within 3-fold compared with 63% when Kp was assumed to be 1 for other tissues (Table 4). However, it improved prediction of compounds with low V_D,ss_. Measured adipocyte and myocyte partition data provided in Supplemental Table 3 enable further exploration of V_D,ss_ prediction methods.

## Conclusions

One of the purposes of comparing various in silico V_D,ss_ prediction methods was to establish the best in silico approaches to predict V_D,ss_ for de novo compounds. Based on the extensive comparisons of results across the in silico methods (Table 2), we conclude that 1) the mechanistic V_D,ss_ prediction methods using a combination of ML models for predicting physicochemical properties paired with mechanistic equations for Kp or 2) the Wajima method employing predicted rat and dog V_D,ss_ are our recommended in silico approaches to predict human V_D,ss_. If a larger training data set of chemically diverse V_D,ss_ experimental values is available, then direct ML predictions of V_D,ss_ might be the most computationally efficient and predictive way to process in silico predictions of V_D,ss_ for de novo compounds. Once these de novo compounds have been synthesized in discovery, it is most useful to experimentally measure BPR and fup to get to a more accurate estimation of human V_D,ss_. Based on our analysis, BPR is the most sensitive physicochemical property to determine V_D,ss_ in silico. Further, we investigated the utility of adipocyte and myocyte partitioning in predicting V_D,ss_. If fat or muscle partition coefficients are being considered as part of the model, adipocyte Kp measurements may provide more predictive power than either myocyte Kp alone or adipocyte and myocyte combined. In summary, the scale of prediction strategies evaluated and size of data sets used in this study are novel and significantly larger than those presented in the literature thus far. In addition, we investigated novel methodologies such as adipocyte and myocyte partitioning in predicting V_D,ss_. Finally, we have provided several novel in vitro data sets (e.g., BPR, adipocyte Kp, myocyte Kp) generated using a single protocol for 254 clinical compounds that will enable the research community to further enhance V_D,ss_ prediction methods.

## Abbreviations

AAFE: absolute average fold error
ATOM: Accelerating Therapeutics for Opportunities in Medicine
BPR: blood-to-plasma partition ratio
CHI: chromatographic hydrophobicity index
ECFP: extended connectivity fingerprint
fup: fraction unbound in plasma
Kp: tissue-to-plasma partition coefficient
LC/MS/MS: liquid chromatography with tandem mass spectrometry
ML: machine learning
MOE: molecular operating environment
PK: pharmacokinetic
RED: rapid equilibrium dialysis
V_D,ss_: volume of distribution at steady state
